# DEVELOPING AND RAPIDLY SCALING AN MHEALTH APP IN A 25-SITE PRAGMATIC TRIAL OF ALIVIADO DEMENTIA CARE IN HOSPICE

**DOI:** 10.1093/geroni/igac059.685

**Published:** 2022-12-20

**Authors:** Abraham Brody, Aditi Durga, Ariel Ford, Shih-Yin Lin

**Affiliations:** HIGN at the NYU Rory Meyers College of Nursing, New York, New York, United States; New York University, New York, New York, United States; New York University Rory Meyers College of Nursing, New York, New York, United States; New York University, New York, New York, United States

## Abstract

Following stakeholder engagement in our pilot phase of a large-scale 2-phase NIA funded pragmatic clinical trial in hospice (HAS-QOL Trial) of Aliviado Dementia Care, we utilized a co-design process to develop over a 6-month period and rapidly scale an mHealth application to assist clinicians in performing best practices in care for persons living with dementia receiving hospice. Throughout the 36 months of intervention period, the app has been utilized by at least 6,580 hospice team members, both to complete training, receive mobile push notification nudges, and: 1) perform 6,682 clinical assessment such as the PAINAD for pain and NPI-Q for behavioral symptoms; 2) develop 2,204 symptom specific care plans; and 3) Review specially developed education materials to 15,128 caregivers and send them to 671 directly. This session will also discuss the process of implementation and scaling, including the technological hurdles in working with a workforce with sometimes limited technical skills.

